# The miR-133b/brefeldin A-inhibited guanine nucleotide-exchange protein 1 (ARFGEF1) axis represses proliferation, invasion, and migration in cervical cancer cells

**DOI:** 10.1080/21655979.2022.2027063

**Published:** 2022-01-20

**Authors:** Lingling Jiang, Xuexin Wang

**Affiliations:** Department of Gynaecology and Obstetrics, First People’s Hospital of Wenling, Wenling, Zhejing, China

**Keywords:** miR-133b, ARFGEF1, proliferation, invasion, migration, cervical cancer

## Abstract

Cervical cancer is a common gynecological malignancy, and miR-133b is an abnormally expressed cervical cancer gene, which suggests that miR-133b may be involved in the occurrence and development of cervical cancer. However, the underlying mechanism is still unclear. miR-133b was overexpressed or silenced in the cervical cancer cell line C33A. Brefeldin A-inhibited guanine nucleotide-exchange protein 1 (ARFGEF1) was combined with overexpression of miR-133b in C33A cells. Cell Counting Kit-8, clone formation, and Transwell assays were performed to determine the influence of miR-133b and ARFGEF1 on clone formation, proliferation, migration, and invasion of C33A cells. The interaction between miR-133b and ARFGEF1 was verified using a luciferase reporter assay. Finally, the mRNA and protein expression of miR-133b and ARFGEF1 in the tumor and adjacent normal tissues of cervical cancer patients was detected by real-time quantitative PCR, Western blotting, and immunohistochemistry. The results indicated that miR-133b up-regulation suppressed the proliferation, invasion, migration, and clone formation abilities of C33A cells (*P* < 0.05). However, silence of miR-133b promoted the proliferation, invasion, and migration of C33A cells (*P* < 0.05). Clone formation ability of C33A cells was also elevated by miR-133b deficiency (*P* < 0.05). Moreover, miR-133b interacted with ARFGEF1 and repressed ARFGEF1 expression in C33A cells (*P* < 0.05). ARFGEF1 overexpression weakened miR-133b overexpression-mediated inhibition of proliferation, invasion, and migration of C33A cells (*P* < 0.05). miR-133b expression was decreased, and ARFGEF1 was up-regulated in tumor tissues of cervical cancer patients (*P* < 0.05). All results revealed that miR-133b suppresses cervical cancer progression by inhibiting proliferation, invasion, and migration of cervical cancer cells via targeting ARFGEF1. Thus, our study determined the mechanism of miR-133b in cervical cancer, and confirmed miR-133b/ARFGEF1 may become a potential therapeutic target for cervical cancer.

## Introduction

Cervical cancer is a common gynecological malignancy with high morbidity and mortality rates and seriously threatens the health of women around the world [1]. 99.7% of cervical cancer patients are detected with high-risk human papillomavirus (HPV) genotypes worldwide, as the persistent viral infection is the major causative agent [2]. Due to precocious puberty and HPV infection, the incidence of cervical cancer is on the rise, especially in the younger generation [3,4]. In recent years, HPV tests and cytological screening have been widely used. This has allowed cervical cancer and precancerous lesions to be detected and treated early [5,6]. HPV vaccines decrease cervical cancer rates 1%–1.9% annually [7]. However, metastasis and the recurrence of cervical cancer are difficult to cure completely, which increases patient mortality. Therefore, clarifying the pathogenesis of cervical cancer has important significance for improving the cure rate of cervical cancer and reducing mortality.

MicroRNA (miRNA) is a type of endogenous non-coding single-stranded RNA, which takes part in various biological processes, such as cell differentiation, tumor growth, and angiogenesis [8,9]. miRNA has a dual role in regulating gene expression. It not only degrades the target mRNA or inhibits protein synthesis, thereby causing gene silencing effects, it can also up-regulate gene expression [10]. Many specifically expressed miRNAs in tumors show oncogene or tumor suppressor gene-like effects [11]. miR-133b has been reported to exert anti-cancer effects in various cancers. Ma et al. revealed that the miR-133b/lung cancer associated transcript 1 (LUCAT1)/enhancer of zeste 2 polycomb repressive complex 2 subunit (EZH2) axis represses metastasis of colorectal cancer [12]. In lung cancer, miR-133b is significantly decreased, and it acts as a tumor repressor to inhibit lung cancer development by inhibiting the SRY-box transcription factor 9 (SOX9)/b-catenin signaling pathway [13]. miR-133b regulates the collagen type 1 alpha 1 chain (COL1A1)/transforming growth factor β (TGF-β) axis and, thus, inhibits cell invasion and migration in gastric cancer [14]. Additionally, miR-133b is also an important molecule in cervical cancer. Chen et al. found that miR-133b is the target of long intergenic non-protein coding RNA 2381 (LINC02381), and LINC02381 accelerates cell viability and migration in cervical cancer by repressing the mRNA expression of miR-133b [15]. The study of Liang et al. confirmed the abnormal expression of miR-133b in cervical cancer, suggesting that miR-133b may be associated with cervical cancer progression.

Brefeldin A-inhibited guanine nucleotide-exchange protein 1 (ARFGEF1) is located mainly in the Golgi apparatus and plays a role in maintaining the structural integrity of the Golgi apparatus [16]. ARFGEF1 participates in regulating the transport of the Golgi apparatus-related vesicles [17]. Previous research has demonstrated that ARFGEF1 functions as a target of miRNAs and takes part in various cancers. For example, miR-215 exerts an anti-cancer effect in papillary thyroid cancer by modulating the epithelial-mesenchymal transition through the inhibition of its target, ARFGEF1 [18]. miR-27b interacts with ARFGEF1 and inhibits ARFGEF1 expression in colon cancers. The miR-27b/ARFGEF1 axis represses colon cancer progression [19]. However, the function of ARFGEF1 in cervical cancer is still unclear. Moreover, the bioinformatics online analysis tools, TargetScan, miRDB, and miRWalk, predicted that miR-133b might bind to ARFGEF1. Thus, we reasonably speculated that miR-133b could regulate cervical cancer progression by targeting ARFGEF1.

In this study, we investigated the effects of miR-133b on the proliferation, invasion, and migration of cervical cancer cell line, C33A, as well as the potential target gene of miR-133b. Furthermore, the role of the miR-133b/ARFGEF1 axis in the progression of cervical cancer was elucidated, providing a theoretical basis for clinical treatment of cervical cancer.

## Materials and methods

### Cell culture

The human cervical cancer cell line C33A (ATCC, Manassas, VA, USA) was cultured at 37°C and 5% CO_2_ in Dulbecco’s modified Eagle medium (DMEM, Invitrogen, San Diego, CA, USA) supplemented with 10% fetal bovine serum (FBS, Invitrogen) and antibiotic solution (Solarbio, Beijing, China).

### Cell transfection

For overexpression or knockdown of miR-133b, the mimics or inhibitors of miR-133b (miR-133b mimic, miR-133b inhibitor), and the negative control (NC) oligonucleotides (mimic NC (NC1), and inhibitor NC (NC2)) were synthesized. For ARFGEF1 overexpression, the full length of ARFGEF1 was synthesized by polymerase chain reaction (PCR) and then subcloned into the pCEP4 vector (pCEP4-ARFGEF1) using conventional methods [20]. The empty pCEP4 vector (pCEP4-NC) was used as a control. These oligonucleotides and vectors were synthesized and obtained from GenePharma (Shanghai, China). C33A cells (1 × 10^5^) were seeded into 24-well plates and then transfected with 2.5 μg of oligonucleotides (miR-133b mimic, NC1, miR-133b inhibitor, or NC2) using Lipofectamine 2000 Transfection Reagent (Invitrogen). C33A cells were co-transfected with pCEP4-ARFGEF1, pCEP4-NC and miR-133b mimic, or NC1. The modified C33A cells were cultured in DMEM supplemented with 300 mg/mL geneticin (Sangon Biotech (Shanghai) Co., Ltd., China). After 48 hours of culture, the modified single colonies were isolated and stored at −20°C for further use.

### Cell proliferation

The ability of C33A cells to proliferate was assessed using the cell counting kit-8 (CCK-8) assay with the protocol described [21]. C33A cells (2000 cells/μL) were cultured in 96-well plates at 37°C and 5% CO_2_. After 12, 24, 48, and 72 hours of culture, 100 μL CCK-8 reagent was incubated with the cells in each well for 1 h at 37°C. The optical density (450 nm) of each sample was detected using a Thermo Multiskan Sky microplate reader (Thermo Fisher Scientific, Waltham, MA, USA).

### Cell migration and invasion

The migration and invasion abilities of C33A cells were detected using the Transwell assay with Transwell plates (BD Falcon, Bedford, MA, USA) [22]. For the invasion assay, Matrigel (Corning, Corning, NY, USA) was loaded into the upper chambers of 24-well Transwell inserts with 8 mm pore polycarbonate filters. The 24-well Transwell chambers without Matrigel were used for migration assays. C33A cells (5 × 10^4^) were seeded into the upper chambers containing DMEM media and 1% FBS. DMEM media containing 10% FBS was added into the bottom chambers. After 48 hours of culture, the invading and migrating cells on the membrane bottom were fixed with 4% paraformaldehyde and stained with 1% crystal violet. Images of the invading and migrating cells were observed under a light microscope (Olympus Optical Co., Ltd., Tokyo, Japan) with five random fields.

### Clone formation

The ability of C33A cells to form clones was assessed. C33A cells were resuspended in DMEM medium to prepare a single‑cell suspension. Cells at a concentration of 300 /well were cultured at 37°C for 2 weeks in a 6-well plate containing DMEM medium. Then, 1% crystal violet was used to stain the cell clones for 30 min. Finally, the number of clones was calculated [23].

### Bioinformatics analysis

The bioinformatics online analysis tools TargetScan (http://www.targetscan.org/vert_72/), miRWalk (http://mirwalk.umm.uni-heidelberg.de/), and miRDB (http://mirdb.org/) were used to predict the prognostic miRNAs that would bind to ARFGEF1. The miRNAs that might bind to ARFGEF1 were intersected with the differentially expressed miRNA between normal cervical tissues and cervical cancer tissues, as previously reported [24]. A Venn diagram was used to overlap the target genes to elevate the reliability of the bioinformatics analysis. Two miRNAs may interact with ARFGEF1, namely miR-133b and miR-548aw. In this study, we verified whether miR-133b could affect cervical cancer development by targeting ARFGEF1.

### Luciferase reporter assay

The wild-type (Wt)/mutant type (Mut)-predicted binding sites between ARFGEF1 and miR-133b were cloned into the vectors pmir-GLO-ARFGEF1-Wt and pmir-GLO-ARFGEF1-Mut (GenePharma), respectively. The pmir-GLO-ARFGEF1-Wt/Mut and miR-133b mimic, and mimic NC (NC1) were transfected into 293 T cells using transfection reagent. A dual luciferase assay kit (Promega, Madison, USA) was used to examine the firefly and Renilla luciferase activities on a luciferase assay system (Ambion, Austin, TX, USA) [25].

### Gene expression

Total RNA Extractor (Trizol) (Sangon Biotech) was used to extract total RNA from C33A cells and tissues. cDNA was synthesized from total RNA applying Superscript II reverse transcriptase (Invitrogen). Real-time quantitative PCR (qRT-PCR) was carried out applying TB Green® Premix Ex Taq™ II (Tli RNaseH Plus) (Takara, Beijing, China) in a Thermal Cycler Dice™ Real Time System series (Takara) following the manufacturer’s instructions. The miR-133b mRNA expression was calculated using the 2^−ΔΔCt^ method [26], with glyceraldehyde-3-phosphate dehydrogenase (GAPDH) as an internal control. The sequences (5’-3’) of the primers were as follows: GAPDH: F (forward): 5’-ACG GAT TTG GTC GTA TTG GG-3’; R (reverse): 5’-TGA TTT TGG AGG GAT CTC GC-3’; miR-133b: F (forward): 5’-GCG AGC ACA GAA TTA ATA CGA CT-3’; R (reverse): 5’-CAC TAT AGG TTT Ranse TTT Ranse CG-3’.

### Protein expression

The protein expression of ARFGEF1 in C33A cells and tissues was assessed by performing Western blotting. The Tissue or Cell Total Protein Extraction Kit (Sangon Biotech) was used to extract total proteins. Subsequently, the equivalent proteins were separated by 10% sodium dodecyl sulfate polyacrylamide gel electrophoresis (SDS-PAGE) and then transferred to a nitrocellulose membrane (Millipore, Plano, TX, USA). The nitrocellulose membranes were stained with rabbit anti-human ARFGEF1 (1:1000 dilution, Abcam, Cambridge, MA, USA) or GAPDH (1:1000 dilution, Abcam) antibodies, and then incubated with goat anti-rabbit horseradish peroxidase (HRP)-IgG (1:2000 dilution, Abcam) antibodies. The immunoreactive bands were visualized with electrochemiluminescence reagents (Millipore). Finally, the Image J software was used to analyze the densitometric readings of the immunoreactive bands [27].

### Clinical sample collection

Cervical cancer patients (n = 30) undergoing cervical cancer surgery in the First People Hospital of Wenling were recruited for this study. Patients with other chronic diseases or a family history of malignancy were excluded from this research. None of the cervical cancer patients were receiving treatments such as radiotherapy or chemotherapy before their surgery. The tumor and adjacent normal tissues were removed from the cervical cancer patients during surgery. These tissues were stored at −80°C or embedded in paraffin for further use. All patients signed consent. The protocols carried out were in accordance with the World Medical Association Declaration of Helsinki and ratified and supervised by the ethics committee of the First People Hospital of Wenling.

### Immunohistochemistry analysis

Immunohistochemistry (IHC) was carried out to measure ARFGEF1 expression in the tumor tissues and adjacent normal tissues from cervical cancer patients [28]. Paraffin sections (5 μm) of the tissues were deparaffinized and hydrated. The sections were treated with citrate antigen retrieval solution (Beyotime Biotechnology) for antigen retrieval and then incubated with 3% H_2_O_2_ to inactivate the activity of the endogenous peroxidase. Subsequently, the tissue sections were stained successively with rabbit anti-human ARFGEF1 (1:100 dilution, Abcam) and HRP-IgG (1:2000 dilution, Abcam). Diaminobenzidine (DAB) and hematoxylin were then used successively to stain the tissue sections. The stained sections were observed under a light microscope.

### Statistical analysis

Each experiment was repeated three times, and representative data are presented. The data are shown as the mean ± standard deviation (SD). Statistical analysis was performed in GraphPad Prism 7 (GraphPad Software, Inc., La Jolla, CA, USA). Statistical differences between the groups were assessed using the Student’s *t*-test and one-way analysis of variance. *P* < 0.05 was considered statistically significant [29].

## Results

To investigate the role of miR-133b in the progression of cervical cancer, and clarify its underlying mechanism, the effects of miR-133b on the proliferation, invasion, and migration of C33A cells were detected. Besides, the target gene of miR-133b was determined by bioinformatics analysis and dual luciferase reporter assay. Moreover, the expression levels of miR-133b and ARFGEF1 were measured in patients with cervical cancer.

### miR-133b inhibited the proliferation, invasion, and migration of cervical cancer cells

**Figure 1.**
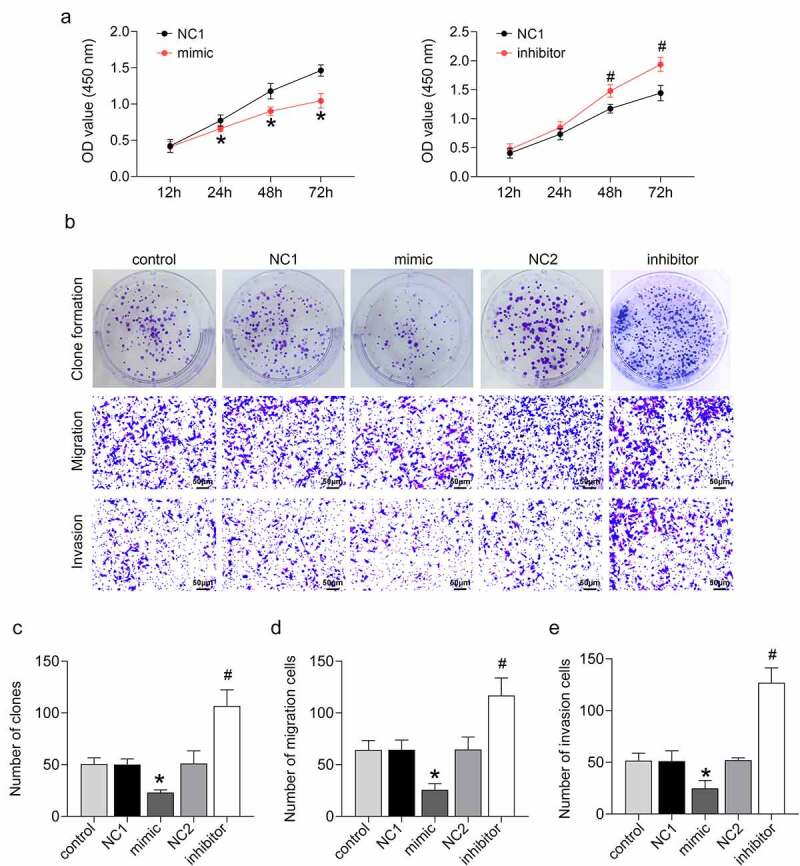
miR-133b inhibited the proliferation, invasion, and migration of C33A cells.

miR-133b was overexpressed or silenced in C33A cells by cell transfection to determine the functional role of miR-133b in cervical cancer. The CCK-8 assay confirmed that miR-133b overexpression significantly repressed cell proliferation in C33A cells (*P* < 0.05). Cell proliferation was notably increased in C33A cells following transfection of the miR-133b inhibitor (Figure 1a, *p* < 0.05). Clone formation in C33A cells showed that miR-133b overexpression caused decreased clone formation in the C33A cells (*P* < 0.05). The number of clones was enhanced in C33A cells in the presence of miR-133b inhibitor (Figure 1(b,c), *P* < 0.05). The Transwell assay revealed that up-regulation of miR-133b suppressed C33A cell migration and invasion, while miR-133b deficiency elevated the migration and invasion ability of C33A cells (Figure 1(b,d,e), *P* < 0.05). Thus, these data imply that miR-133b represses the proliferation, invasion, migration, and clone formation ability of cervical cancer cells.

C33A cells were transfected with miR-133b mimic, NC1, miR-133b inhibitor, NC2. (a) Cell proliferation was detected using the CCK-8 assay. (b) Images of clone formation, migration, and invasion. (c) Clone formation was detected. (d–e) Migration and invasion were measured using Transwell assays. *P < 0.05 vs. NC1; ^#^*P* < 0.05 vs. NC2.

### miR-133b interacted with ARFGEF1 in cervical cancer cells

**Figure 2.**
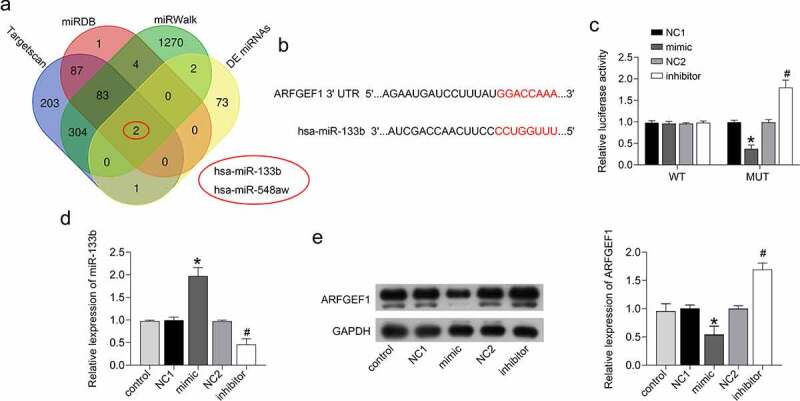
miR-133b interacted with ARFGEF1 in C33A cells.

The bioinformatics online analysis tools TargetScan, miRDB, and miRWalk predicted the prognostic miRNAs that would bind to ARFGEF1. Further analysis found that miR-133b and miR-548aw may interact with ARFGEF1 (Figure 2a). These two miRNAs are also abnormally expressed in cervical cancer patients, as previously reported. Here, we verified whether miR-133b could affect the development of cervical cancer by targeting ARFGEF1. The luciferase reporter assay demonstrated that miR-133b interacted with ARFGEF1 (Figure 2(b,c)). We further estimated the impact of miR-133b on the protein expression of ARFGEF1 in C33A cells. The mRNA expression of miR-133b was notably increased in the C33A cells after transfection of miR-133b mimic (*P* < 0.05). Deficiency of miR-133b led to pronounced down-regulation of miR-133b in C33A cells (Figure 2d, *p* < 0.05). We also found that ARFGEF1 protein expression in C33A cells was repressed by miR-133b overexpression and enhanced by miR-133b knockdown (Figure 2e, *p* < 0.05). Therefore, these findings confirmed that miR-133b affects ARFGEF1 expression in cervical cancer cells by targeting ARFGEF1.

(a) The overlapping target miRNAs of ARFGEF1 were predicted using TargetScan, miRDB, and miRWalk. Venn diagram of miRNAs. (b) The predicted binding sites between ARFGEF1 and miR-133b. (c) The interaction between miR-133b and ARFGEF1 was verified through luciferase reporter assays. C33A cells were transfected with miR-133b mimic, NC1, miR-133b inhibitor, NC2. miR-133b and ARFGEF1 expression were measured by qRT-PCR (d) and Western blotting (e). *P < 0.05 vs. NC1; ^#^*P* < 0.05 vs. NC2.

### Overexpression of ARFGEF1 weakened the influence of miR-133b on the proliferation, invasion, and migration of cervical cancer cells

**Figure 3.**
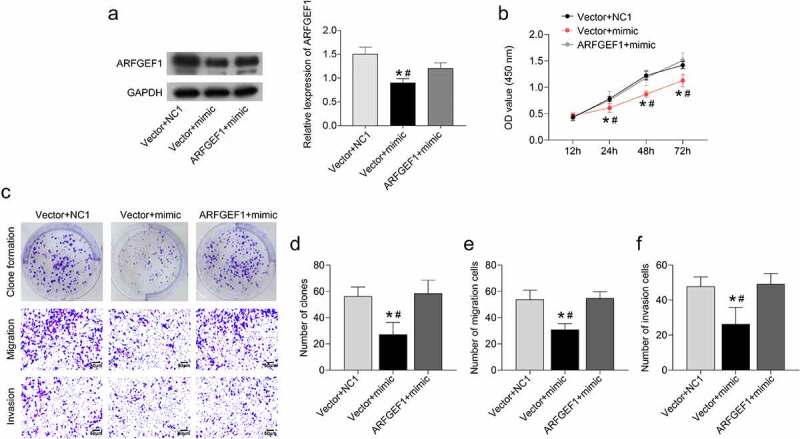
Overexpression of ARFGEF1 weakened the impact of miR-133b on proliferation, invasion, and migration of C33A cells.

We further verified the underlying mechanism of the miR-133b/ARFGEF1 axis in cervical cancer. Up-regulation of miR-133b repressed ARFGEF1 protein expression in C33A cells (*P* < 0.05). However, ARFGEF1 overexpression weakened miR-133b up-regulation-mediated inhibition of ARFGEF1 protein expression in C33A cells (Figure 3a, *p* < 0.05). Moreover, up-regulation of miR-133b inhibited C33A cell proliferation, which was abolished by ARFGEF1 overexpression (Figure 3b, *p* < 0.05). Clone formation of C33A cells was decreased following transfection of miR-133b mimic (*P* < 0.05). ARFGEF1 up-regulation abrogated the inhibiting effect of miR-133b overexpression on the clone formation ability of C33A cells (Figure 3(c,d), *P* < 0.05). Additionally, the migration and invasion abilities of C33A cells were reduced by miR-133b overexpression (*P* < 0.05). The miR-133b up-regulation-mediated inhibition of migration and invasion was abolished by ARFGEF1 overexpression (Figure 3(c,e,f), *P* < 0.05). Therefore, overexpression of ARFGEF1 weakens the influence of miR-133b on the proliferation, invasion, and migration ability of cervical cancer cells.

C33A cells were co-transfected with miR-133b mimic, NC1, and pCEP4-ARFGEF1, pCEP4-NC. (a) ARFGEF1 expression was measured by Western blotting. (b) Cell proliferation was detected using the CCK-8 assay. (c) Images of clone formation, migration, and invasion. (d) Clone formation was detected. (e–f) Migration and invasion were measured using Transwell assays. *P < 0.05 vs. Vector + NC1; ^#^*P* < 0.05 vs. ARFGEF1 + mimic.

### miR-133b expression was reduced and ARFGEF1 expression was elevated in patients with cervical cancer

**Figure 4.**
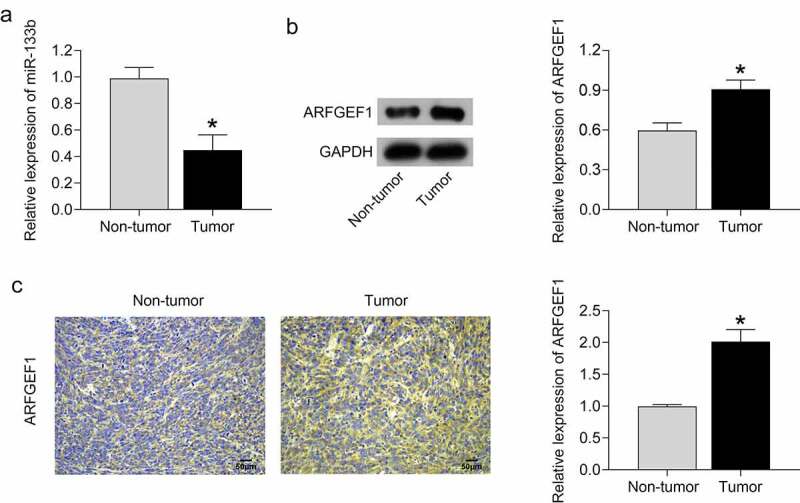
MiR-133b expression was decreased and ARFGEF1 expressed was increased in patients with cervical cancer.

Finally, we compared the expression of miR-133b and ARFGEF1 between tumor tissues and adjacent normal tissues in cervical cancer patients. miR-133b expression was severely decreased in tumor tissues compared with adjacent normal tissues (Figure 4a, *p* < 0.05). As shown in Figure 4(b,c), using Western blotting and IHC assays, the tumor tissues of cervical cancer patients exhibited an up-regulation of ARFGEF1 protein with respect to adjacent normal tissues (*P* < 0.05). Thus, we confirmed that tumor tissues of cervical cancer patients display a down-regulation of miR-133b and an up-regulation of ARFGEF1.

(a) The mRNA expression of miR-133b in the tumor tissues and adjacent normal tissues was estimated using qRT-PCR. (b–c) The protein expression of ARFGEF1 in tumor tissues and adjacent normal tissues was estimated by Western blotting and immunohistochemistry (IHC) analysis. *P < 0.05 vs. Non-tumor.

## Discussion

miR-133b was originally discovered to be specifically expressed in muscle tissues, and is highly expressed during the development of human skeletal muscle [30]. Actually, miR-133b is widely expressed in a range of tissues and plays important functions. For example, abnormal expression of miR-133b was discovered in mouse granular cells, and increases the expression of forkhead box L2 (Foxl2)-mediated transcription of steroid hormone-related genes steroidogenic acute regulatory protein (StAR) and cytochrome p450 family 19 subfamily A member 1 (CYP19A1) by directly targeting Foxl2. Thus, it promotes the secretion of estrogen from granulosa cells [31]. miR-133b also regulates the differentiation of nerve cells and is related to the pathogenesis of Parkinson’s, a neurodegenerative disease [32]. Additionally, much research has revealed that miR-133b is abnormally expressed in cervical cancer patients, suggesting that miR-133b is related to cervical cancer [24,33]. LINC02381 accelerates the metastasis of cervical cancer cells through the regulation of the miR-133b/Ras homolog family member A (RhoA) axis [34]. In this study, we detected the levels of miR-133b in tumor tissues and adjacent normal tissues, and the results revealed that miR-133b level was reduced in cervical cancer patients, which verified the previous research findings. Then, we further determined the mechanism of miR-133b in cervical cancer. miR-133b overexpression exhibited inhibitory effects on proliferation, invasion, migration, and clone formation in cervical cancer cells. However, a deficiency of miR-133b resulted in a severe decrease in the proliferation, invasion, migration, and clone formation ability in cervical cancer cells. Thus, miR-133b may exert a tumor suppressor effect in cervical cancer.

Accumulating research has determined the function of ARFGEF1 in regulating the function of neurons. Miyamoto et al. confirmed that ARFGEF1 initiates myelination by Schwann cells, and ARFGEF1 deficiency in Schwann cells decreases the thickness of myelin in mice [17]. Inhibition of ARFGEF1 reduces the tissue size of the neocortex and hippocampus, impedes neuronal polarization, and promotes neuron apoptosis, thereby affecting neuron survival in the developing embryonic brain [35]. Furthermore, ARFGEF1 also has a crucial role in various cancers. miR-376 c acts as a tumor suppressor in osteosarcoma by repressing proliferation, invasion, and differentiation via Wnt family member 3 (Wnt-3) and ARFGEF1 signaling pathways [36]. Herein, we revealed the interaction between miR-133b and ARFGEF1 in cervical cancer, as both bioinformatics online analysis and dual luciferase reporter assay confirmed that ARFGEF1 was the direct target gene of miR-133b. miR-133b inhibited ARFGEF1 protein expression in cervical cancer cells, and overexpression of ARFGEF1 weakened the inhibitory effects of miR-133b on the proliferation, invasion, migration, and clone formation ability of C33A cells, suggesting miR-133b/ARFGEF1 axis repressed biological functions of cervical cancer cells Moreover, we verified that ARFGEF1 expression was severely elevated in patients with cervical cancer. Compared with previous studies [37,38], our study found a new pathway related to cervical cancer, and provided new biomarkers of the cervical cancer progression.

## Conclusion

In summary, this study revealed the anti-cancer effects of miR-133b in cervical cancer. miR-133b targets ARFGEF1 to inhibit proliferation, invasion, and migration in cervical cancer cells. Thus, this work reveals the molecular mechanism of the miR-133b/ARFGEF1 axis in cervical cancer and suggests novel insight for the treatment of cervical cancer.

## Highlights

1. miR-133b inhibited proliferation, invasion, and migration in cervical cancer cells.
2. miR-133b interacted with ARFGEF1.
3. Overexpression of ARFGEF1 weakened the effects of miR-133b.
4. Cervical cancer patients exhibited a downregulation of miR-133b and an upregulation of ARFGEF1.
